# An Uncommon Culprit: Dural Arteriovenous Fistula Presenting as Otodynia and Dizziness

**DOI:** 10.7759/cureus.63602

**Published:** 2024-07-01

**Authors:** Rima El Atrache, Anit K Behera, Lintu Ramachandran, Kasra Rahbar

**Affiliations:** 1 Neurology, Baylor College of Medicine, Houston, USA; 2 Stroke, Baylor College of Medicine, Houston, USA; 3 Neurocritical Care, Baylor College of Medicine, Houston, USA; 4 Interventional Neuroradiology, Michael E. DeBakey Veterans Affairs Medical Center, Houston, USA

**Keywords:** dizziness, diagnostic imaging, ear pain, davf, arteriovenous fistulas

## Abstract

Dural arteriovenous fistulas (DAVFs) are rare vascular abnormalities that can present with diverse neurological symptoms. We report a case of a woman in her early 60s who presented with pain in the left ear and dizziness. Neurological evaluation and imaging studies revealed a DAVF in the left cerebellopontine angle. This case underscores the importance of considering DAVF as a potential etiology in patients presenting with atypical otological symptoms.

## Introduction

Dural arteriovenous fistulas (DAVFs) are characterized by abnormal shunting across the dural layer, connecting arteries directly to the sinus, meningeal, or cortical veins without an intervening capillary network. These malformations are typically acquired and often idiopathic, although sometimes associated with a prior history of venous thrombosis, trauma, or craniotomy. They may pose a risk of intracranial hemorrhage and non-hemorrhagic neurological deficit (NHND) due to venous congestion, accounting for their morbidity and mortality [1,2].

DAVFs are rare, accounting for less than 15% of intracranial vascular malformations. These are more prevalent in females than males, close to 60 years of age [1]. DAVFs commonly involve the transverse-sigmoid sinus, superior sagittal sinus, tentorium, anterior cranial fossa, and cavernous sinus (indirect carotid-cavernous fistula). Arterial feeders are most often from branches of the external carotid artery (e.g. occipital or middle meningeal arteries), but can also arise from internal carotid artery branches (e.g. meningohypophyseal trunk), or from meningeal branches of other intracranial arteries (e.g. posterior cerebral, superior cerebellar, or vertebral arteries).

The range of clinical presentation varies from asymptomatic, for incidental DAVFs, to acute presentation from intracranial hemorrhage, and progressive subacute or chronic presentation from NHND due to venous congestion. Headache and pulsatile tinnitus are common symptoms, although not specific or present in all cases. Pulsatile tinnitus is caused by turbulent blood flow in proximity to the temporal bone. Other neurologic manifestations include ocular symptoms, sensory and motor changes, decreased level of consciousness, cognitive decline, seizures, and elevated intracranial pressure, depending on anatomical location and patterns of venous outflow [3,4]. The Borden and Cognard systems are two classification systems used to categorize DAVFs according to venous drainage, with implications for management and prognosis. The presence of cortical venous drainage (CVD), corresponding to higher Borden and Cognard grades, is associated with an elevated risk of aggressive behavior, particularly if CVD is clinically symptomatic [5].

Hearing loss, tinnitus, and vestibular dysfunction are occasionally reported. There are a few documented cases of ipsilateral otalgia with posterior condylar canal and transverse sigmoid sinus DAVFs, with resolution of otalgia and other symptoms after embolization [6-9]. Otological symptoms as the primary manifestation of DAVF are rare, posing diagnostic challenges. We present a case of DAVF mimicking inner ear pathology to highlight the importance of considering vascular etiologies in patients with atypical ear symptoms.

## Case presentation

A woman in her early 60s presented with a seven-month history of daily left inner ear pain, left neck tension, and intermittent dizziness. The ear pain, described as intermittent, aching, non-radiating, and localized to the inner ear, was rated 6/10 and exacerbated by weightlifting and ipsilateral arm elevation. She described dizziness as a sensation of imbalance with lightheadedness. She reported left-sided pulsatile tinnitus for several years, which resolved spontaneously a year before presentation and several months prior to the onset of otological symptoms.

**Figure 1:**
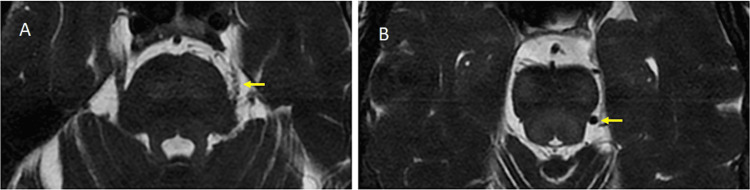
MRI T2 SPACE sequence of the cerebellopontine angles (A) The image shows vascularity along the surface of the left trigeminal nerve; (B) The image shows a dilated left lateral mesencephalic draining vein MRI: Magnetic resonance imaging; SPACE: Sampling perfection with application-optimized contrasts using different flip angle evolution

**Figure 2:**
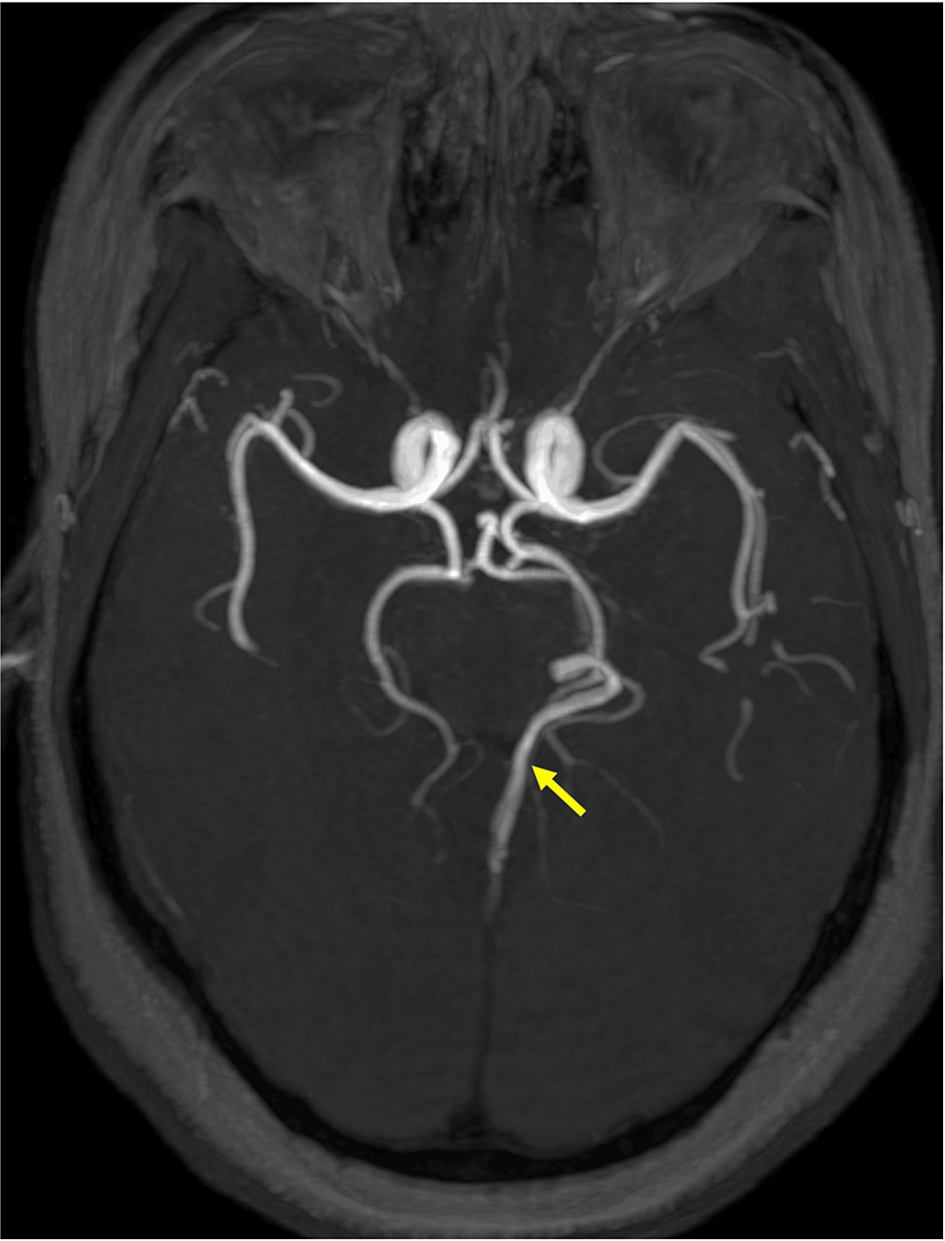
MRA of the left lateral mesencephalic vein Axial MIPs of time-of-flight MRA demonstrating flow-related enhancement of the left lateral mesencephalic vein, draining into the vein of Galen MIPs: Maximum intensity projections; MRA: Magnetic resonance angiography

**Figure 3:**
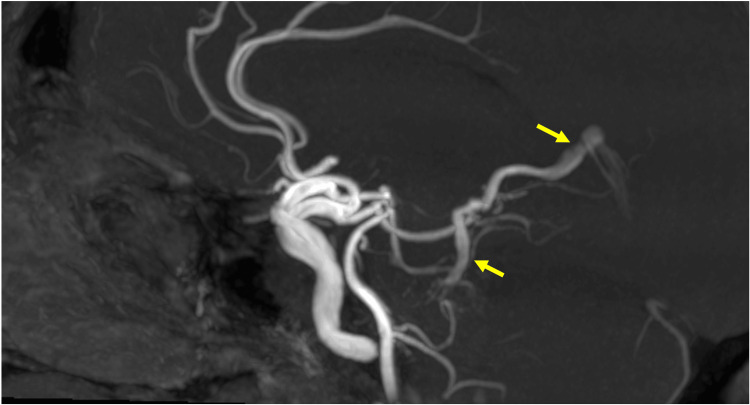
MRA of the left lateral mesencephalic vein Sagittal MIPs of time-of-flight MRA demonstrating flow-related enhancement of the left lateral mesencephalic vein, draining into the vein of Galen MIPs: Maximum intensity projections; MRA: Magnetic resonance angiography

MRI brain with internal auditory canal (IAC) protocol and magnetic resonance angiography (MRA) head and neck revealed a focal hypervascularity in the left cerebellopontine angle (CPA), in contiguity with the left trigeminal nerve suspicious of a DAVF supplied by the left superior cerebellar artery branches and left posterior cerebral artery and draining via a single collector vein into the left perimesencephalic vein and vein of Galen (Figures 1-3).

**Figure 4:**
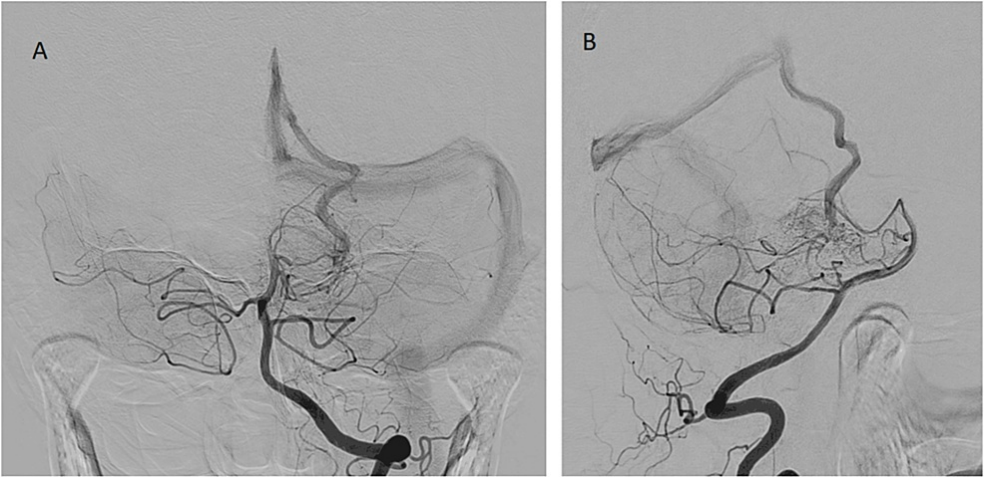
Digital subtraction angiography (A) DSA in AP and (B) lateral projections demonstrating a 2 cm loose arterial network supplied by branches of the left SCA and PCA and shunting into the lateral mesencephalic vein DSA: Digital subtraction angiography; AP: Antero-posterior; SCA: Superior cerebellar artery; PCA: Posterior cerebral artery

**Figure 5:**
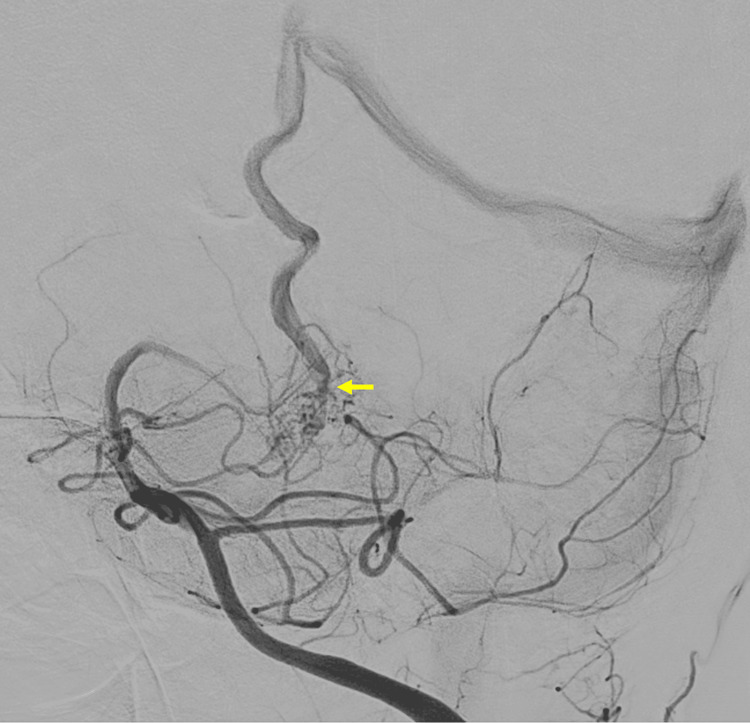
Digital subtraction angiography Magnified LAO projection demonstrating angioarchitecture of the arterial network and drainage via a single collector vein from a single fistulous point (arrow) LAO: Left anterior oblique

Catheter angiogram demonstrated the angioarchitecture of arterial feeders and drainage into a single fistulous locus (Figures 4-5). Embolization was offered, but the patient declined, opting for conservative management and watchful waiting.

## Discussion

This case underscores the diagnostic challenge posed by DAVF presenting with atypical otological symptoms. While inner ear pain and dizziness are uncommon manifestations, they should prompt consideration of vascular abnormalities, especially in the presence of associated neurological signs. In this case, the provocation of otological symptoms with weightlifting and ipsilateral arm elevation, which transiently elevates venous pressures, as well as the historical presence of ipsilateral pulsatile tinnitus, may be clinical clues of a vascular etiology. The spontaneous resolution of pulsatile tinnitus one year before the presentation and several months before the onset of otological symptoms may indicate a change in the venous drainage pathway that precipitated the development of otological symptoms.

DAVFs can occur in various locations within the intracranial space, with the CPA being an uncommon but notable site [10,11]. The trigeminal nerve innervates an extensive sensory area in the head and is a common channel of referred otalgia. It provides sensory innervation to the face and is divided into three main branches, with motor innervation to the muscles of mastication. In the ear, the auriculotemporal branch helps supply parts of the auricle, ear canal, and middle ear [12]. The trigeminal nerve is not a common site for DAVF involvement, and its association with inner ear symptoms adds complexity to the diagnostic challenge. DAVFs close to the trigeminal nerve can cause symptoms of ipsilateral facial pain [10,13,14]. The presence of inner ear pain and dizziness in our patient may be attributed to the proximity of the DAVF to the trigeminal nerve, potentially causing irritation or compression of adjacent neural structures. 

Diagnosing DAVFs may be challenging, given the diverse range of presenting symptoms and sometimes subtle or absent findings on cross-sectional imaging. Diagnostic imaging, including MRI with an IAC protocol and MRA, played a crucial role in identifying the DAVF and delineating its anatomical features. The focal hypervascularity observed in the left CPA, contiguous with the trigeminal nerve, provided important clues to the underlying pathology. Catheter angiography is the gold standard for diagnosing and characterizing DAVFs, especially when cross-sectional imaging modalities may be negative. Early recognition and treatment can prevent potential complications such as intracranial hemorrhage or neurological deficits in DAVFs associated with CVD. Treatment decisions depend on anatomical features, cortical venous drainage, and symptom severity. Many DAVFs are treatable with endovascular techniques, depending on anatomical location. Surgical management remains a mainstay for lesions that are not safely amenable to endovascular treatment. Radiosurgery is a second-line treatment modality due to its moderate efficacy and long latency period. Regardless of the treatment approach, disconnection of the arterial inflow from the venous outflow is necessary for a cure.

In this case, the presence of drainage by a brainstem vein warrants consideration of treatment. Transarterial embolization via the superior cerebellar or posterior cerebral arterial feeders is unsafe due to the risk of non-target embolization. Transvenous embolization and surgical management were considered as potential options and discussed with the patient. Ultimately, the patient opted for watchful waiting, given the consequences of ischemic or hemorrhagic complications in this location with either endovascular or surgical treatment. When patients are managed conservatively, they should undergo repeat angiographic imaging if new symptoms develop [15]. 

## Conclusions

This case report highlights a rare presentation of a DAVF in the left CPA, contiguous with the trigeminal nerve, presenting with atypical otological symptoms. Clinicians should be aware of atypical symptoms caused by DAVFs and the role imaging modalities play in diagnosis and characterization.
